# A platform-independent AI tumor lineage and site (ATLAS) classifier

**DOI:** 10.1038/s42003-024-05981-5

**Published:** 2024-03-13

**Authors:** Nicholas R. Rydzewski, Yue Shi, Chenxuan Li, Matthew R. Chrostek, Hamza Bakhtiar, Kyle T. Helzer, Matthew L. Bootsma, Tracy J. Berg, Paul M. Harari, John M. Floberg, Grace C. Blitzer, David Kosoff, Amy K. Taylor, Marina N. Sharifi, Menggang Yu, Joshua M. Lang, Krishnan R. Patel, Deborah E. Citrin, Kaitlin E. Sundling, Shuang G. Zhao

**Affiliations:** 1 Radiation Oncology Branch, National Cancer Institute, National Institutes of Health, Bethesda, MD USA; 2 Department of Human Oncology, University of Wisconsin, Madison, WI USA; 3 Carbone Cancer Center, University of Wisconsin, Madison, WI USA; 4 Department of Medicine, University of Wisconsin, Madison, WI USA; 5 Department of Biostatistics and Medical Informatics, University of Wisconsin, Madison, WI USA; 6 Department of Pathology and Laboratory Medicine, University of Wisconsin, Madison, WI USA; 7 Wisconsin State Laboratory of Hygiene, University of Wisconsin, Madison, WI USA; 8 William S. Middleton Veterans Hospital, Madison, WI USA

**Keywords:** Cancer genomics, Cancer of unknown primary, Metastasis, Prognostic markers, Molecular medicine

## Abstract

Histopathologic diagnosis and classification of cancer plays a critical role in guiding treatment. Advances in next-generation sequencing have ushered in new complementary molecular frameworks. However, existing approaches do not independently assess both site-of-origin (e.g. prostate) and lineage (e.g. adenocarcinoma) and have minimal validation in metastatic disease, where classification is more difficult. Utilizing gradient-boosted machine learning, we developed ATLAS, a pair of separate AI Tumor Lineage and Site-of-origin models from RNA expression data on 8249 tumor samples. We assessed performance independently in 10,376 total tumor samples, including 1490 metastatic samples, achieving an accuracy of 91.4% for cancer site-of-origin and 97.1% for cancer lineage. High confidence predictions (encompassing the majority of cases) were accurate 98–99% of the time in both localized and remarkably even in metastatic samples. We also identified emergent properties of our lineage scores for tumor types on which the model was never trained (zero-shot learning). Adenocarcinoma/sarcoma lineage scores differentiated epithelioid from biphasic/sarcomatoid mesothelioma. Also, predicted lineage de-differentiation identified neuroendocrine/small cell tumors and was associated with poor outcomes across tumor types. Our platform-independent single-sample approach can be easily translated to existing RNA-seq platforms. ATLAS can complement and guide traditional histopathologic assessment in challenging situations and tumors of unknown primary.

## Abstract (web summary)

ATLAS is a pair of AI Tumor Lineage and Site-of-origin machine learning models, that can accurately classify both primary and metastatic tumors using high-throughput RNA expression data and can identify de-differentiated anaplastic tumors.

## Introduction

Histopathologic assessment has been the primary modality for the diagnosis of human cancers since the 19^th^ century, and to this day remains the mainstay of diagnosis, risk stratification and staging. While the field has made countless advances, the art of pathology relies heavily on subjective visual inspection, with considerable levels of inter-observer variability in diagnosis^1–3^, which can impact treatment decisions. Tumors are molecularly complex and even pathologic specimens that appear visually similar may have widely different clinical behaviors. Furthermore, the origin of metastatic tumors is sometimes difficult to ascertain using traditional histopathologic approaches due to heterogenous features or tumor de-differentiation. Immunohistochemistry, in situ hybridization, as well as other techniques have emerged to augment morphology alone, and are routinely used clinically in identification of both the site of origin (e.g. prostate, breast, lung) and cancer lineage (e.g. adenocarcinoma, squamous cell cancer (SCC), etc.). However, there is a limit on how many stains can be applied, requiring a priori selection. Furthermore, the number of pathologists in the US has decreased by 18% between 2007 and 2017, while cancer cases have increased by 17%, which has yielded a 41% increase in workload for pathologists^4^. This shortage can greatly impact cancer care unless new methodologies to assist pathologists can be implemented. In recent decades, next generation sequencing (NGS) of DNA, RNA, and the epigenome have transformed our understanding of the alterations that define and drive carcinogenesis. NGS represents an extension of the histologic techniques described above and can be thought of as an indirect microscopy at the molecular scale. Rather than relying on fluorescence and visual assessment to identify and quantify macromolecules, quantitative NGS approaches can capture molecular features that are undetectable visually.

NGS and other -omics techniques have exponentially increased the amount of data collected on cancer patients over the past decade, and numerous commercial assays are now used in the clinic. Interpretation of this quantity of data poses its own challenges, and computational techniques such as machine learning (ML) have emerged to turn data into useful clinical tools. However, the utility of these clinical tools depends strongly on the datasets on which the classifier is validated, and which clinical features of the tumors can be identified. While there are published tissue of origin prediction tools available, they lack sufficient validation on metastatic samples and neglect the critical diagnostic component of independent assessment of site of origin and cancer lineage. These models rely on a diverse range of data on which to train a classifier, such as DNA alterations^5–8^, DNA methylation^9–13^, and mRNA^14–23^ or microRNA^24^ expression. DNA alterations (mutation status, copy number alteration (CNA)) are widely assessed, but unfortunately, many oncogenes and tumor suppressors are altered across multiple cancer types, which can be a limiting factor of mutation-based cancer of origin ML models^5–7^. Despite these limitations, ML models using DNA alterations have achieved accuracies up to 88% across 24 cancer types on independent validation^6^. DNA methylation is an epigenomic alteration that regulates gene expression, with certain alterations being highly cancer type specific. Most DNA methylation ML models have only been validated in small institutional cohorts or in hold-out test sets, not true independent validation cohorts, limiting our ability to assess their generalizability. Expression of certain mRNAs and microRNAs have also been found to be tumor type specific, and ML models built on the expression of each have been shown to be highly accurate. One large study (TOD-CUP)^23^ achieved an accuracy in independent validation of 91% across 4 cancer types in 1029 TCGA microarray samples, 94% across 4 cancer types in 2277 non-TCGA primary tumor samples, and 94% accuracy across 5 cancer types in 141 metastatic samples. A more recent deep learning-based model^15^ achieved an accuracy in independent validation of 91.4% across 18 cancer types in 2085 samples from the ICGC dataset, including an accuracy of 88.1% in 395 metastatic samples. While these results represent an improvement over DNA alteration-based strategies, the vast majority are validated in primary tumor samples, with limited data on performance in metastatic samples, where site of origin is likely more difficult to predict due to tumor evolution and de-differentiation. In addition, cancer lineage (e.g. adenocarcinoma vs SCC) is a critical component of diagnosis and treatment planning but is often left out or paired with the site of origin, rather than being assessed as an independent axis.

In this study, we created AI Tumor Lineage and Site (ATLAS) classifiers, trained on NGS from 8249 samples, that predict cancer site of origin (22 classes) as well as cancer lineage (8 classes). This independent classification is distinct from prior studies and improves clinical utility. This bi-modular framework allows for separate evaluation of both important axes, for a total of 176 different possible combinations, and allows evaluation of lineage de-differentiation into more anaplastic or neuroendocrine forms. We then independently assessed the performance of our models on 10,376 tumor samples, including 1490 metastatic samples, the largest such validation of an expression-based classifier to date, especially in metastatic disease. In addition, our single-sample approach is platform-independent and agnostic to how the sample was collected and processed, producing accurate and interpretable predictions that can be applied to any existing RNA-seq platform. As tumor RNA-seq becomes routine, this tool can be readily integrated into pathologic clinical decision-making and provide objective and quantitative orthogonal information to help guide pathologic diagnosis.

## Results

### Modeling workflow and data overview

**Fig. 1:**
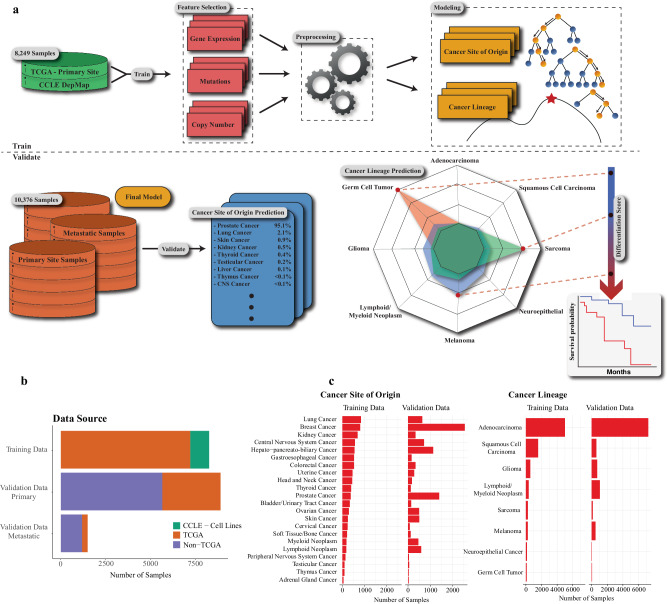
Modeling workflow and data overview. Modeling workflow **a** depicts the primary workflow for model building—data partitioning (training versus validation), training data feature selection (determine best sequencing data and model features to build an effective model), data pre-processing (such as normalizing expression data and imputing missing values), and model selection. Once an optimal model is selected using only the training data, a validation dataset is used to validate the locked model. The training data included the Cancer Genome Atlas (TCGA) and Cancer Cell Line Encyclopedia (CCLE) cell line samples. Validation was completed on over 10,000 patient samples, including over 1400 metastatic samples (**b** – TCGA orange, CCLE green, non-TCGA purple). Two models were built – a cancer site of origin model with 22 classes and a cancer lineage model with 8 classes (**c**), with validation samples for all classes.

To build the most comprehensive genomic classifier of cancer site of origin and lineage to date (Fig. 1a) we utilized 8249 samples from the Cancer Genome Atlas Program (TCGA, *N* = 7196) and the Cancer Cell Line Encyclopedia (CCLE, *N* = 1053) for ATLAS model training. The validation cohort consisted of 10,376 total samples, including 58 TCGA datasets (*N* = 3556, none overlapping with the training data) and 41 additional non-TCGA datasets (*N* = 6820). This included validation in primary tumors (*N* = 8886 from 97 datasets) and in metastatic tumors (*N* = 1490 from 17 datasets). The final training and validation cohorts included 22 cancer site of origin classes and 8 cancer lineage classes (Fig. 1b, c). Since many different RNA-seq platforms were used across datasets, each sample was independently normalized^25^ with no required batch correction, allowing for a more clinically useful per patient normalization strategy. All training samples had gene expression data, mutation calls, and copy number alteration calls, which allowed for a comparison of each molecular feature in model building.

### Accurate predictions of cancer site of origin and lineage

**Fig. 2:**
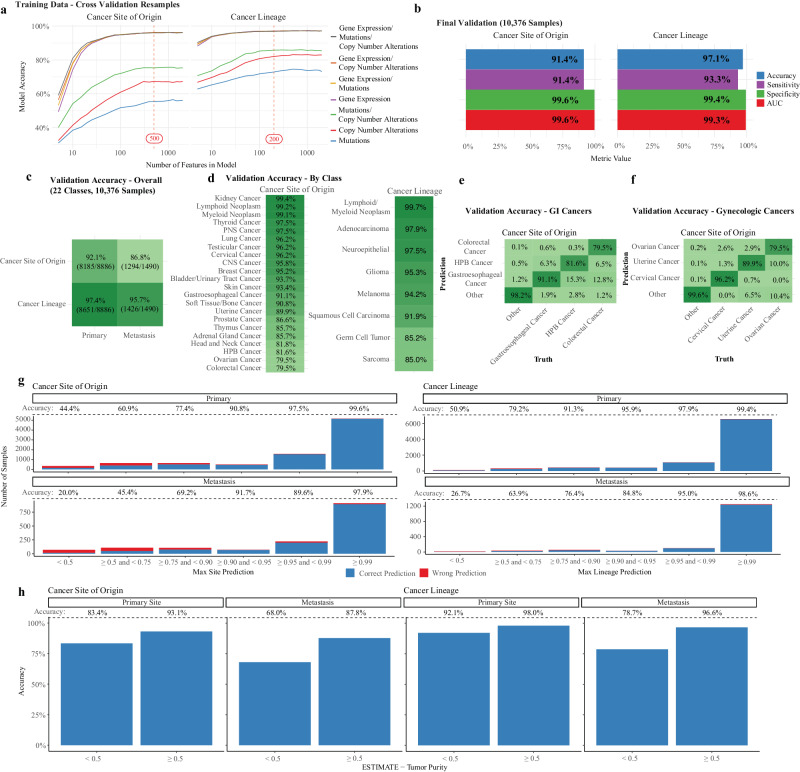
Accurate predictions of cancer site of origin and lineage. Cancer site of origin and cancer lineage models were trained and performance was evaluated on five-fold cross-validation resamples, noting top performance with gene expression (**a** – gene expression/mutations/copy number alterations grey, gene expression/copy number alterations orange, gene expression/mutations yellow, gene expression purple, mutations/copy number alterations green, copy number alterations blue, mutations blue, with order of legend matching position of curves), with no improvement when combined with mutation and copy number calls. The finalized models included 500 features and 200 features for the cancer site of origin and cancer lineage models, respectively. Model validation accuracy on 10,376 samples was 91.4% for the cancer site of origin model and 97.1% for the cancer lineage model (**b** – accuracy blue, sensitivity purple, specificity green, AUC is red). Performance for these models was worse on metastatic samples, but still very high accuracy at 86.8% and 95.7%, respectively (**c** – darker shades of green represent higher accuracy). Model accuracy by predicted class is shown (d), noting worse performance in gastrointestinal (GI) sites and gynecologic sites (**e**-**f**). All model prediction classes had a corresponding probability score, with the maximum score corresponding to the predicted class. When the probability score was ≥ 0.99 (a majority of samples in all sub-groups shown), the models had very high accuracy (**g** – correct prediction red, wrong prediction blue). When samples were stratified by low tumor purity (ESTIMATE Tumor Purity < 0.5), accuracy of both models was found to be higher in samples with a high tumor purity (**h**). AUC is the area under the receiver operating characteristic (AUC-ROC) curve. HPB Cancer – Hepato-pancreato-biliary cancer.

The first step of our workflow was to train separate models to predict for cancer site of origin and cancer lineage. We first evaluated the importance of different molecular features (i.e. gene expression, mutation, and copy number) and impact of the total number of molecular features in model performance (Fig. 2a). We assessed these two questions in our training data by using a 5-fold cross validation (CV) re-sampling schema (detailed in the methods). With regards to molecular feature type, we found that mutation status alone, copy number alone, or the combination of the two performed worse in CV than any combination that included gene expression. Since gene expression seemed to perform just as well alone as adding DNA alterations, we moved forward with a model using only gene expression. With regards to the number of features, CV performance increased initially as the number of features was increased, but plateaued for site of origin at around 500 features (including a binary sex variable) and lineage at around 200 features (only genes), which were used for the final models (detailed in methods). There was some overlap of genes between the two models (68 genes), but overall, the majority of genes in both models were unique and contributed to a final model framework that required only 632 features.

The performance of these two models (comprising ATLAS) were then assessed in the independent validation cohort (Fig. 2b). Overall accuracy was 91.4% for cancer site of origin and 97.1% for cancer lineage (*N* = 10,376). However, there was a large difference in accuracy for site of origin between primary tumors (92.1%, comparable to prior studies; *N* = 8886) vs. metastatic tumors (86.8%; Fig. 2c; *N* = 1490). This difference is unsurprising given that the models were trained on primary tumors, in addition to tumor evolution and de-differentiation that occurs with progression to metastatic disease. Interestingly, the difference in performance for cancer lineage was less (97.4% in primary tumors vs. 95.7% in metastatic samples). While overall accuracy was high, gastro-intestinal (GI) and gynecologic (GYN) tumors tended to have worse classification accuracy (Fig. 2d). GI tumors were commonly mis-classified as other GI tumors, with hepato-pancreato-biliary (HPB) tumors (*N* = 1128) mis-classified as gastroesophageal in 15% of cases, and colorectal tumors (*N* = 337) mis-classified as gastroesophageal tumors in 13% of cases (Fig. 2e). For GYN tumors, 10% of ovarian tumors (*N* = 498) were mis-classified as uterine tumors (Fig. 2f). To understand the impact of the binary sex variable on accuracy for the cancer site of origin model, all validation samples were run through the model with sex imputed as missing, resulting in a drop of accuracy from 91.4% to 90.7%. The benefit of including sex was primarily driven by improved accuracy in a subset of breast cancer (*N* = 45), ovarian cancer (*N* = 19), and cervical cancer (*N* = 7) samples. Median Shapley values^26^ for each class prediction were obtained from the training data to identify the features that had the largest influence determining the site of origin and lineage classes, providing the top 10 features for each class in Supplementary Data 1. To confirm that features were specific to the tumor and not just normal tissue, we also report the top 10 features for each class among correctly predicted metastatic samples. This analysis confirmed that the sex variable was only a top feature in breast, ovarian and cervical cancer, and further identified many well-validated and novel markers that can be used to differentiate tumor types.

Overall, the strength of the model prediction correlated well with the accuracy (Fig. 2g). For both site of origin and lineage, if the classifier prediction was ≥0.99 (encompassing 58.5% of the validation samples for cancer site, 75.1% of the validation samples for cancer lineage), this correlated with a 98–99% accuracy, even in metastatic samples. The correlation between model confidence and accuracy is important in interpreting the predictions and differentiating between high-confidence cases versus more equivocal ones. Finally, samples with low tumor purity (<50%, calculated by ESTIMATE^27^) have worse accuracy compared to those with high purity (Fig. 2h) for both primary samples (*N* = 931 low purity samples; 10.5% of primary samples) and metastatic samples (*N* = 75 low purity samples; 5.0% of metastatic samples).

### Accurate distinction between adenocarcinoma versus SCC lineage across cancer sites

**Fig. 3:**
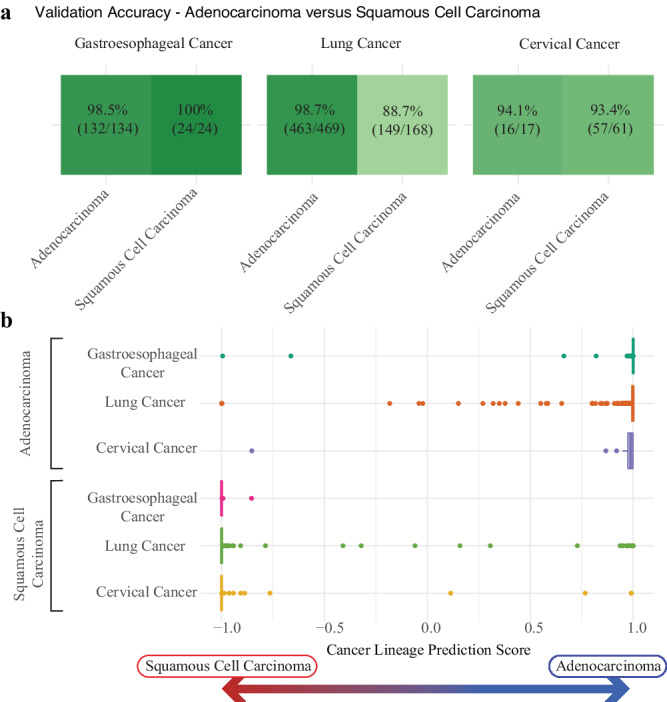
Accurate distinction between adenocarcinoma versus SCC lineage across cancer sites. Focusing on all cancer sites that had at least 10 adenocarcinoma and squamous cell carcinoma (SCC) samples—gastroesophageal cancer (134 adenocarcinomas, 24 SCCs), lung cancer (469 adenocarcinomas, 168 SCCs), and cervical cancer (17 adenocarcinomas, 61 SCCs)—the cancer lineage model maintained highly accurate predictions for all subtypes (**a** - darker shades of green represent higher accuracy). Each sample has both a squamous cell carcinoma probability score and an adenocarcinoma probability score. The difference between these two scores is plotted **b**, showing that the vast majority of samples had scores corresponding strongly to the appropriate cancer lineage. For the presented boxplots, boxes show the interquartile range, encompassing the middle 50% of the data, the median is indicated by a line within the box, whiskers extend to 1.5× the interquartile range, and points beyond this are plotted as outliers.

Cancer site of origin and lineage are often intertwined. For example, tumors of the breast are predominantly adenocarcinomas, whereas tumors of the head & neck are predominantly SCC. For some sites, tumors can arise from either an adenocarcinoma or SCC lineage, a difference that is important to identify as it can impact treatment decisions. In order to ensure that our lineage classifier was accurately distinguishing lineage (as opposed to indirectly measuring it by predicting site), we further examined the accuracy at predicting cancer lineage stratified by cancer site of origin, specifically focusing on adenocarcinoma vs. SCC. In our validation dataset, three tumor sites (gastroesophageal, lung, cervix) had relatively large numbers (≥10) of both adenocarcinoma and SCC tumors, including 134 adenocarcinoma and 24 SCC gastroesophageal cancers, 469 adenocarcinoma and 168 SCC lung cancers, and 17 adenocarcinoma and 61 SCC cervical cancers. Overall, the accuracy of our lineage classifier in distinguishing between adenocarcinoma and SCC was high, ranging from 89% to 100% across the three sites (Fig. 3a). When we looked at the difference between the adenocarcinoma and SCC lineage scores, we saw clearly separate distributions between adenocarcinomas and SCCs (Fig. 3b).

### Sarcomatoid differentiation in mesothelioma

**Fig. 4:**
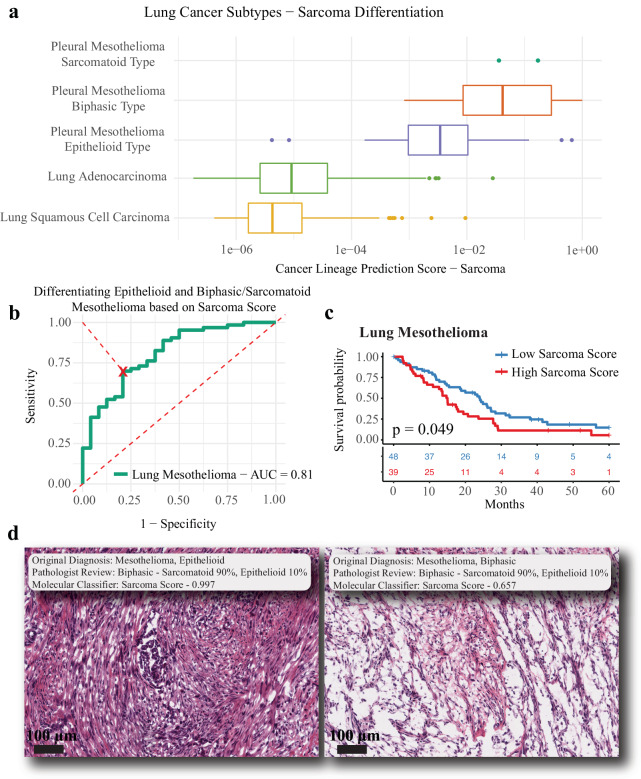
Sarcomatoid differentiation in mesothelioma. The sarcoma cancer lineage score was evaluated further in mesothelioma samples to determine the models ability to identify subtypes that were not present in model training. The sarcoma score was higher in pleural mesothelioma samples (sarcomatoid type [*N* = 2; no boxplot shown], biphasic type [*N* = 23], and epithelioid type [*N* = 63]) compared to non-small cell lung cancer samples (Wilcoxon rank-sum test *P* < 0.001; adenocarcinoma [*N* = 469] and squamous cell carcinoma [*N* = 168]), and also was higher in mesothelioma biphasic/sarcomatoid subtypes compared to epithelioid subtypes (Wilcoxon rank-sum test *P* < 0.001; **a**. The continuous sarcoma score was effective in differentiating epithelioid pleural mesothelioma samples from biphasic/sarcomatoid mesothelioma samples (AUC = 0.81, **b**). To create binary sarcoma score groups, an optimal cut-point was identified in the lung mesothelioma ROC curve (red X in **b**) that minimized the distance to the point where sensitivity and specificity were both one. These binary sarcoma score groups were prognostic for lung mesothelioma samples (logrank *P* = 0.043; **c** – low sarcoma score blue [*N* = 48], high sarcoma score red [*N* = 39]; dotted red line represents a null AUC of 0.5). Our in-house pathologist reviewed the two pathologic specimen that had the highest sarcoma scores to compare our molecular classification score against pathologic review (intermediate magnification, measuring bar represents 100 µm; **d**. For the presented boxplots, boxes show the interquartile range, encompassing the middle 50% of the data, the median is indicated by a line within the box, whiskers extend to 1.5× the interquartile range, and points beyond this are plotted as outliers. AUC is the area under the receiver operating characteristic (AUC-ROC) curve.

Mesothelioma of the lung is a pleural-based tumor that arises from the mesothelium, commonly due to exposure to asbestos. This tumor type is unique in having three distinct subtypes, epithelioid, sarcomatoid (more aggressive), and biphasic (a mix of the epithelioid and sarcomatoid). Thus, it serves as an excellent tumor type in which to study the distribution of the sarcoma lineage score. Given the small total number of lung mesothelioma samples available (*N* = 88), we decided to remove all mesothelioma samples from TCGA and all other cohorts, excluding them from any of the training or validation thus far. Thus, the sarcoma lineage predictions would be made in a tumor type that the model was never trained on (termed Zero Shot Learning or ZSL^28^). In these 88 lung mesothelioma samples, we first examined the distribution of the sarcoma lineage scores across subtypes, as well as comparing them to non-small cell lung cancer (NSCLC) tumor lineages (adenocarcinoma and SCC). The sarcoma lineage scores were higher in mesothelioma (*N* = 88, median = 0.0065) compared to the other NSCLC tumor types (*N* = 637, median = 0.000007; Wilcoxon rank-sum test *P* < 0.001; Fig. 4a). Within mesothelioma, the sarcomatoid and biphasic subtypes (*N* = 25) have higher sarcoma lineage scores (median = 0.042) compared to the epithelioid subtype (*N* = 63, median=0.003; Wilcoxon rank-sum test *P* < 0.001; Fig. 4a). The sarcoma lineage score had a high area under the receiver operating characteristic (AUC-ROC) curve for differentiating epithelioid samples from biphasic/sarcomatoid samples (AUC = 0.81; Fig. 4b). An optimal cut was identified and used to create high and low sarcoma lineage score groups, which were prognostic for survival (Fig. 4c, log-rank *P* = 0.049), with a median survival of 15.0 months and 23.9 months, respectively. The sarcoma lineage score results are remarkably consistent with the known phenotypic subtypes of mesothelioma, revealing an emergent property of our lineage models on which the model was not directly trained in an example of ZSL. Two mesothelioma samples had such high sarcoma lineage scores that they were classified as sarcoma by our model. In the original pathology data, one of these samples was reported as biphasic, and the other as epithelioid. We performed blinded re-review of the TCGA histological images by an institutional pathologist, who described both samples as biphasic with approximately 90% sarcomatoid differentiation (Fig. 4d). These cases illustrate the potential clinical utility of our molecular classifier. Divergence in initial pathologic review and strong molecular classifier results could suggest re-review or additional stains.

### De-differentiated lineage associated with neuroendocrine disease

Tumors unfortunately do not remain static as they progress from primary to metastatic tumors and evolve under various selective pressures such as treatment. De-differentiation into more anaplastic tumors is a well-established phenomenon across cancer types^29^. Neuroendocrine differentiation is a specific example of this, associated with a more aggressive phenotype in prostate cancer^30,31^ and lung cancer^32,33^. We characterized the degree of differentiation by focusing on the cancer lineage model predictions for this analysis, which would produce eight cancer lineage scores for each sample. Each sample will have a maximum cancer lineage score, which we collected and labeled as a “differentiation score”. The rationale behind this categorization was that a weaker resemblance towards a particular lineage indicates a more de-differentiated and anaplastic tumor.

**Fig. 5:**
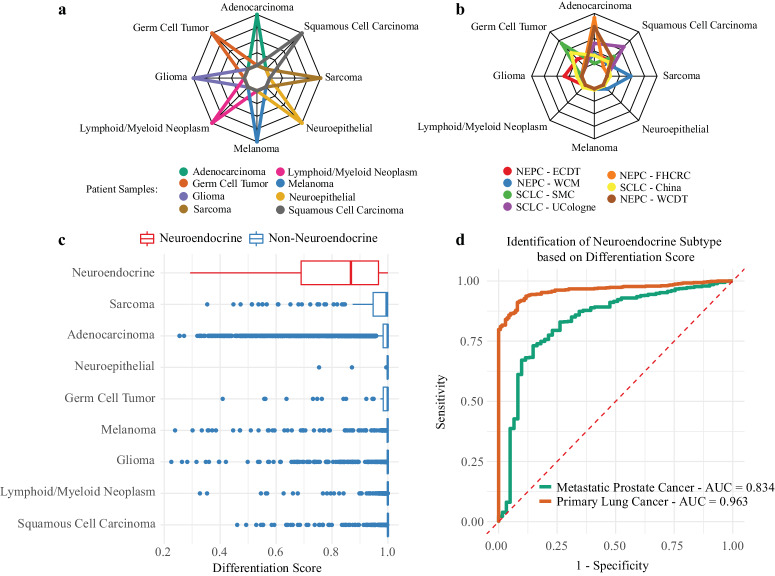
De-differentiated lineage associated with neuroendocrine disease. For every sample the cancer lineage model produced 8 prediction scores to correspond to the 8 lineage subtypes. The results of selected samples from the validation cohort were then plotted on a radar plot to evaluate the heterogeneity of prediction scores. Most samples had very strong predictions for a single lineage subtype **a**. Neuroendocrine samples, including neuroendocrine prostate cancer (NEPC) and small cell lung cancer (SCLC), had more heterogenous predictions **b**, noting that the max probability was lower in these samples compared to non-neuroendocrine samples. This max prediction probability (Differentiation Score) was compared across all samples and noted that neuroendocrine samples had lower scores when compared to all other samples (Wilcoxon rank-sum test *P* < 0.001; **c**—neuroendocrine red, non-neuroendocrine blue; Neuroendocrine [*N* = 198], Sarcoma [*N* = 147], Adenocarcinoma [*N* = 7256], Neuroepithelial Cancer [*N* = 40], Germ Cell Tumor [*N* = 54], Melanoma [*N* = 501], Glioma [*N* = 718], Lymphoid/Myeloid Neoplasm [*N* = 1054], and Squamous Cell Carcinoma [N = 606]). This continuous differentiation score was then evaluated for its ability to differentiate metastatic prostate adenocarcinoma samples (PRAD) from NEPC samples (AUC = 0.833) and differentiate non-small cell lung cancer samples (NSCLC) from SCLC samples (AUC = 0.963; **d** metastatic prostate cancer green [N = 782], primary lung cancer orange [*N* = 743]; dotted red line represents a null AUC of 0.5). For the presented boxplots, boxes show the interquartile range, encompassing the middle 50% of the data, the median is indicated by a line within the box, whiskers extend to 1.5× the interquartile range, and points beyond this are plotted as outliers. AUC is the area under the receiver operating characteristic (AUC-ROC) curve. WCM Weill Cornell Medicine, SMC Samsung Medical Center, FHCRC Fred Hutchinson Cancer Research Center, ECDT East Coast Dream Team, UCologne University of Cologne, WCDT West Cost Dream Team.

We first evaluated the performance of this differentiation score in identifying malignant neuroendocrine tumors. Because TCGA does not include neuroendocrine samples, no neuroendocrine tumors were included in model training, or any of the validation results up to this point. Therefore, we identified an additional 198 neuroendocrine samples (neuroendocrine prostate cancer and small cell lung cancer) from 8 cohorts. The distribution of lineage scores for 8 selected highly differentiated tumors showed very confident predictions for a single cancer lineage (Fig. 5a). This is in contrast with selected neuroendocrine samples, that exhibited de-differentiation towards a more heterogenous distribution of lineage scores with a lower maximum score (Fig. 5b), supporting our rationale for the differentiation score. We noted a clear global decrease of the differentiation score in neuroendocrine tumors (*N* = 198, median = 0.868) compared to non-neuroendocrine lineages (*N* = 10,376, median = 0.999; Wilcoxon rank-sum test *P* < 0.001; Fig. 5c). The differentiation score produced a high ROC AUC (Fig. 5d) for differentiating non-small cell lung cancer (*N* = 606; NSCLC) from small cell lung cancer (*N* = 137; SCLC; AUC 0.963) and for differentiating metastatic prostate adenocarcinoma (*N* = 721) from neuroendocrine prostate cancer (*N* = 61; NEPC; AUC 0.834), representing another example of ZSL with an emergent property of the lineage model on which it was never directly trained.

### De-differentiated lineage associated with worse survival across cancers

**Fig. 6:**
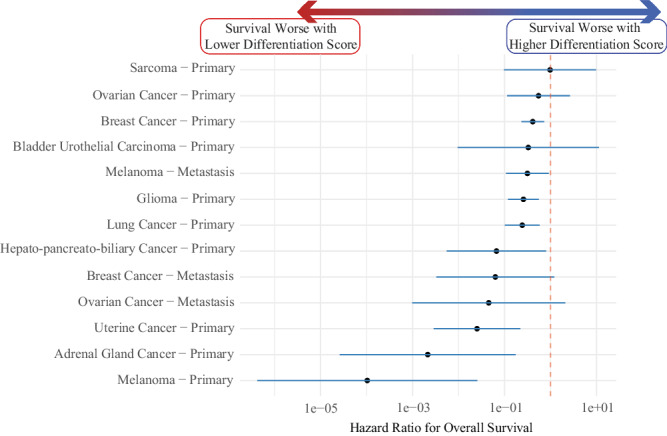
De-differentiated lineage associated with worse survival across cancers. Samples with survival data in the validation cohort were stratified based on their cancer site of origin and biopsy site (primary versus metastatic; primary sarcoma [*N* = 100], primary ovarian cancer [*N* = 356], primary breast cancer [*N* = 2182], primary bladder urothelial carcinoma [*N* = 108], metastatic melanoma [*N* = 399], primary lung cancer [N = 611], primary glioma [*N* = 615], primary hepato-pancreato-bilary cancer [*N* = 424], metastatic breast cancer [N = 121], metastatic ovarian cancer [*N* = 25], primary uterine cancer [*N* = 170], primary adrenal gland cancer [*N* = 26], and primary melanoma [*N* = 88]). Eight of the subgroups evaluated had significantly improved survival with increasing differentiation score, while all other subgroups trended in that direction. Hazard Ration on x-axis has been log10 adjusted. Dotted vertical red line represents a hazard ratio of 1. The black dots represent the hazard ratios and error bars represent the 95% confidence interval for the hazard ratio.

In addition to neuroendocrine differentiation, tumors can also de-differentiate into more anaplastic tumors that are thought to be more aggressive^29,34^. Therefore, we hypothesized that de-differentiation broadly measured by a lower differentiation score would confer worse outcomes across cancer types. We examined all datasets with overall survival data, focusing on subgroups with sufficient samples in each survival outcome group (≥10) and enough variance in the differentiation score (≥0.001). Given that metastatic samples would be expected to have lower differentiation scores, we stratified samples into subgroups based on the cancer site of origin and primary versus metastatic site of biopsy. A reduction in differentiation score results in a significant decrease in the hazards ratio (HR) across eight subgroups (primary melanoma [*N* = 88; HR 0.0001; *P* = 0.001], adrenal [*N* = 26; HR 0.002; *P* = 0.006], uterine [*N* = 170; HR 0.025; *P* = 0.001], HPB [*N* = 424; HR 0.056; *P* = 0.023], glioma [*N* = 615; HR 0.26; *P* = 0.0006], lung [*N* = 611; HR 0.24; *P* = 0.001], breast [*N* = 2182; HR 0.41; *P* = 0.002] and metastatic melanoma [*N* = 399; HR 0.31; *P* = 0.033]), with all other subgroups trending in the same direction (Fig. 6; primary bladder urothelial carcinoma [*N* = 108; HR 0.33; *P* = 0.54], ovarian [*N* = 356; HR 0.55; *P* = 0.45], sarcoma [*N* = 100; HR 0.98; *P* = 0.98], and metastatic ovarian [*N* = 25; HR 0.045; *P* = 0.11] and breast [*N* = 121; HR 0.063; *P* = 0.066]). The association of a more de-differentiated/anaplastic phenotype with worse outcomes is another emergent property of the lineage differentiation scores, highlighting the unique benefits of evaluating lineage separately from site of origin.

## Discussion

Herein, we developed ATLAS, a 22-class cancer site of origin classifier and 8-class cancer lineage classifier trained in 8249 tumor samples. RNA expression using ~600 genes appeared to distinguish site of origin and lineage better than DNA alterations, consistent with the literature^6,16,23^. Interestingly, we show that DNA alterations can be used to build models that perform quite admirably, particularly when using both variant mutations and copy number alterations, but these alterations do not provide any additional information beyond what is captured by RNA expression. We show that the RNA expression classifiers achieve 91.4% accuracy for site of origin and 97.1% accuracy for lineage on a validation dataset of 10,376 tumor samples, the largest and most comprehensive validation of an expression-based classifier to our knowledge. This accuracy is particularly impressive given the wide range of RNA-seq techniques used across the validation data from TCGA and 41 other cohorts, indicating that our approach is truly platform-independent. Histopathologic assessment continues to be the gold standard for diagnosing cancer site of origin and cancer lineage. However, NGS methods could be used to augment histopathology. In cases where it is challenging to determine the primary, a NGS method could help guide the immunohistochemical workup, resolve conflicting staining results, and provide additional information in otherwise unclassifiable cases. Beyond improving accuracy in cases where there is uncertainty, this method can also quantitate the degree of uncertainty.

No approach to classify tumor types is perfect, either histopathology or NGS-based, and variability will always be present^1–3^. In both cases, an assessment of the confidence of the classification is critical in the interpretation of results. In clinical practice, pathologists routinely indicate when diagnosis is uncertain, or should be interpreted with caveats, such as scant tissue, high levels of necrosis or treatment effect, or unclear staining patterns^35^. A challenge of machine-learning NGS approaches is that the final prediction can seemingly come out of a black box (i.e. without comprehensible mechanistic detail), especially in more complex models^36^. Therefore, it is critical that the model predictions themselves contain information on the strength of those predictions in order to provide context for interpretation. An inaccurate prediction is obviously not optimal, but a confidently inaccurate prediction is far worse. A major strength of our classifier is the correlation between accuracy and the prediction score itself (ranging from 0 to 1). The highest scoring and thus most confident predictions, representing the majority of predictions, achieve remarkable accuracies of 98–99%, even in metastatic samples. As the scores and confidence falls, the prediction accuracy also decreases, but this is a quantifiable and reportable result. A physician therefore is able to interpret a low-confidence score of 0.5 very differently than a high-confidence score of 0.99. Future work can explore how such an approach can be incorporated into diagnostic workflows and aid pathologists.

Another unique strength of our approach is the separation of site of origin and lineage into separate classifiers. While the two are certainly related, many sites can give rise to multiple tumor lineages. Both site and lineage ultimately contribute to the final tumor phenotype, and thus we felt it was critical to examine lineage separately. Our classifier accurately distinguishes between different lineages even within the same site (e.g. gastroesophageal, lung, cervix), and is capable of zero-shot learning, identifying sub-lineages in tumor types on which the model was never directly trained (e.g. mesothelioma, neuroendocrine prostate, and small cell lung cancer). Perhaps the most interesting emergent behavior of our model is the ability to identify more de-differentiated or anaplastic tumors, that have concomitantly worse survival across cancer sites of origin. Lineage differentiation is not fixed, and plasticity is a well described phenomenon across cancer types, especially for adenocarcinomas transitioning to aggressive neuroendocrine tumors in prostate and lung primaries^37^. To our knowledge, this is the first pan-cancer signature of lineage de-differentiation and anaplasia that is also integrated into a tumor site of origin and lineage classifier.

While the majority of pathology reports offer clear identification, a substantial 35% are reported by oncologists to contain ambiguous language^38^. While less common, a still substantial 1–2% of cancers are cancers of unknown primary, which presents treatment challenges for clinicians^39^. With RNA-seq of tumors becoming more integrated into standard clinical NGS assays, the platform-independent classifiers we describe herein could complement traditional pathologic assessment, especially in more challenging cases. The ability to globally quantify confidence levels in predicting cancer site of origin, lineage, and tumor de-differentiation is particularly useful, providing a more reproducible quantitative measure than traditional histopathology. These models can continue to be refined as new datasets become available, especially for rare tumor types not currently well represented. The results from such a tool could easily be added to existing clinical RNA-seq reports, complementing traditional histopathologic assessment in cancer research, clinical trial design, and ultimately clinical practice.

## Methods

### Data collection and organization

To develop the models included in this study we sought out a variety of large cancer databases for training and validation—the Cancer Genome Atlas Program (TCGA)^40,41^, the Cancer Cell Line Encyclopedia (CCLE)^42^, the International Cancer Genome Consortium (ICGC)^43^, and cBioPortal^44,45^. Given the standardized format of data located in cBioPortal, we downloaded the TCGA data and most validation datasets from there, while the CCLE, ICGC, and pan-cancer analysis of advanced and metastatic tumors (POG570) data^46^ were downloaded from their respective organizational repositories. We focused only on samples that had RNA expression data available, utilizing DNA mutation and copy number data from the TCGA training cohort only to compare these molecular features against RNA expression in cross-validation.

The goal of our workflow was to predict both cancer site of origin and cancer lineage. Given the heterogeneity of the datasets and understanding that too many classes can result in poor predictions, we consolidated the model classes into 22 cancer site of origin classes and 8 cancer lineage classes **(**Fig. 1). Of note, the neuroepithelial class for the cancer lineage model represents paragangliomas/pheochromocytomas. Primary site (non-metastatic) samples from the TCGA Pan-Cancer Atlas^41^ and samples from the CCLE^42^ were used for model training. Any sample in the CCLE or validation cohort that did not match a cancer subtype in the TCGA Pan-Cancer Atlas dataset was removed (*N* = 531). Lung mesothelioma samples represented a small cohort of samples in the TCGA and likewise would be a useful cancer type to validate the cancer lineage model scores on, and so all lung mesothelioma samples were removed from the primary training and validation cohort. Neuroendocrine prostate cancer (NEPC) and small cell lung cancer (SCLC) samples were not present in the TCGA and so were not part of the validation cohort, but we did use these samples as part of a secondary analysis to evaluate de-differentiation. These secondary analyses on mesothelioma, NEPC and SCLC samples allowed for an evaluation on how the cancer lineage model performed on data that was not included in training.

To produce more accurate and generalizable models we utilized both patient samples (TCGA) and cell lines (CCLE) in the training set to help overcome some of the limitations of both datasets—patient samples from the TCGA will have some non-tumor related normal tissue present that can confound training, in contrast to cell lines which lack this normal tissue but unfortunately will also lack a tumor microenvironment. The training set included 1053 CCLE samples and 75% of the primary site samples from the TCGA Pan-Cancer Atlas (*N* = 7196). The validation dataset included the remaining 25% of the primary site TCGA Pan-Cancer atlas samples, older TCGA samples that did not overlap with the Pan-Cancer atlas, all metastatic TCGA samples, and novel samples downloaded from the ICGC, cBioPortal and POG570 (*N* = 10,376). Validation focused on adult malignancies and, in addition to 58 TCGA validation datasets^40,41^, produced a cohort of 39 independent primary site datasets^30,43,46–71^ and 15 independent metastatic datasets^30,43,46,64–72^. To increase the number of metastatic samples for validation we also included samples from the west coast dream team (WCDT) metastatic prostate cancer dataset that were reported as adenocarcinoma^73,74^. The secondary analysis of neuroendocrine differentiation included SCLC and NEPC samples from 5 studies in cBioPortal^30,69–71,75^, POG570 dataset^46^, the WCDT^73,74^, and an additional dataset of SCLC samples from Jiang et al. ^76^.

### Sequencing data processing

The sequencing data utilized in this workflow included RNA expression, mutations status, and copy number alteration. The RNA expression training data focused on datasets that were not gene-normalized (not Z-score adjusted), and thus RNA expression validation datasets that only included such data were removed. There was a lot of heterogeneity in the per sample normalization schemes used on the expression data, including RSEM, FPKM, RPKM, TPM, CPM, and TMM, including some microarray datasets, with high model accuracy present across normalization schemes. To account for these differences we ran a second normalization on all samples, prior to training and validation, utilizing a per sample Yeo-Johnson transformation^25^ that aims to create a normalized distribution for each sample. This step was sufficient for model training and validation and no further batch correction was required.

DNA data were evaluated only in training to compare to the accuracy of expression-based models. DNA mutation data was filtered to include only coding mutations and was turned into a binary classification (mutant/wildtype). Copy number alteration data was translated into a ternary call (copy number loss, no copy number change, and copy number gain).

### Model building

Data from the TCGA Pan-Cancer Atlas and CCLE were combined into a single group for model building. Sex was included in the cancer site of origin model, and no other clinical variables were included. We first filtered the feature set of both models to include 12,247 genes by removing those with missing expression values and those with the 10% lowest median expression. This gene set was then used to train 6 models—models based on RNA expression, DNA mutations, and copy number alterations that separately predicted the cancer site of origin and cancer lineage. We then ran our modeling workflow (XGBoost, described further below) and optimized the number of trees hyper-parameter based on a five-fold cross validation (CV) re-sampling schema. Hyper-parameter optimization based on the CV resamples identified the best six models, which we then proceeded to evaluate with a model variable importance function^77^ to rank genes in order of most to least important for the model (producing a different rank for the 6 models). This rank list was then utilized to determine how many features would be included in the final models (across a range of 5-2000 features, which include the binary sex variable for the cancer site of origin model). This produced the results in Fig. 2a, which allowed us to identify RNA-seq expression as sufficient for model building and likewise evaluate the minimum number of features required to create an optimal model. We first selected the expression-based cancer of origin and cancer lineage models that produced the best accuracy (1000 features for both models), evaluated the re-sampling 95% confidence interval of that accuracy, and then followed the curve in Fig. 2a to identify the first feature count to fall within that 95% confidence interval (500 features for the cancer of origin model and 200 features for the cancer lineage model). This step was essential to prevent overfitting to the training set and to allow for a more efficient modeling procedure, as a model with more features would take longer to run (for model training, imputing missing values on validation, and making predictions on validation).

### Model validation

The locked in cancer site of origin and cancer lineage models were then evaluated for their performance on the validation dataset. Some samples in the validation cohort had missing values, and so we performed a k-nearest neighbors’ imputation (k = 5) so there would be no missing values when a sample was fed into the model. While the model used (XGBoost) can handle missing values, we observed a validation accuracy of 86.7% with no imputation, compared to our reported accuracy of 92.5% with imputation. Each sample prediction produced 22 probability scores for the cancer site of origin and 8 probability scores for the cancer lineage, with each score corresponding to a class, the scores adding up to one within each model, and a class call produced based on the highest probability score for that sample. The class prediction was utilized to evaluate performance of the models on validation. The probability scores were utilized to evaluate confidence in a prediction and define a differentiation score that was equal to the maximum cancer lineage score for a sample.

### Statistics and reproducibility

All data collection and analysis were performed on our lab Linux server, which included 120 CPU cores, 2 TB of RAM and a single NVIDIA Tesla T4 15GB GPU. All workflow was completed in R (version 4.3.2) and utilized the cBioPortalData package for downloading cBioPortal data^78^, tidyverse^79^, tidymodels^80^, vip^77^, survminer^81^, and fmsb^82^ packages. The model procedure utilized the extreme gradient boosting (XGBoost) machine learning model^83^, which brings together the concepts of decision trees, ensemble learning and gradient boosting into one unified, efficient and highly accurate framework. We utilized XGBoost for all our modeling workflow as it tended to produce similar/improved accuracy on the CV training resamples compared to a random forest model and was able to run significantly faster and utilize our servers GPU. The only hyperparameter in the XGBoost model that we tuned was the number of trees, which we optimized with a five-fold CV scheme.

For our modeling workflow we utilized the area under the receiver operating characteristic curve (AUC), sensitivity, specificity, and accuracy to evaluate the model. Sensitivity and specificity for multi-class classification utilized a one-versus-all, macro-averaging scheme^80^. The AUC that is reported for all multi-class problems represents the Hand-Till method for multiclass classification problems^84^. For the secondary analysis evaluating the continuous cancer lineage sarcoma probability scores in mesothelioma, we developed the binary classes based on the ROC curve of the continuous score and found the split with the minimum distance from the ROC curve to the point where specificity and sensitivity are both one. This cut was determined only on the best split to separate epithelioid lung mesothelioma samples from biphasic/sarcomatoid samples (Fig. 5). Given that this split was not based on optimizing a split in survival, there was no data leakage in creating these prognostic groups. Prognostic significance was evaluated based on overall survival utilizing the Kaplan-Meier estimator and logrank p-values for the mesothelioma sarcoma groups and the Cox regression hazard ratios with 95% confidence intervals for the differentiation score forest plot. All survival data was censored at 5 years to allow for similar comparisons.

### Reporting summary

Further information on research design is available in the Nature Portfolio Reporting Summary linked to this article.

### Supplementary information

Description of Additional Supplementary Files

Supplementary Data 1

Reporting Summary

## Data Availability

Data were primarily downloaded from cBioPortal (https://www.cbioportal.org/datasets), unless otherwise specified. The Cancer Cell Line Encyclopedia (CCLE) dataset were downloaded from the CCLE website (https://sites.broadinstitute.org/ccle/datasets). The West Coast Dream Team (WCDT) data is available at dbGAP (phs001648). The POG570 dataset were downloaded from the British Columbia Genome Sciences Center database (https://www.bcgsc.ca/downloads/POG570/). The Jiang et al. SCLC data were downloaded from the Gene Expression Omnibus (GSE60052). All datasets used in this analysis were previously published and had the appropriate ethical approval for sample collection and publication. Relevent data labels and predictions utilized to create all figures are provided on the ATLAS GitHub page (github.com/nickryd/ATLAS) and minted at Zenodo^85^.

## Code availability

The finalized model workflow and model weights are available at GitHub (github.com/nickryd/ATLAS) and minted at Zenodo^85^. The publicly available model has independent Cancer Site of Origin and Cancer Lineage XGBoost model parameters. The available workflow can take as input a table of samples, where the rows are individual samples and the columns are individual model features (genes and binary sex). The input can be expression from a microarray or RNAseq with any normalization schema, just no per gene normalization across a whole cohort. The model treats each sample independently, first performing a Yeo-Johnson transformation of the gene expression across a sample, conversion of the binary sex variable into a dummy variable, k-nearest neighbor imputation of missing values, and finally a center and scaling of each variable based on parameters determined during model training. A prediction is then made using the locked XGBoost model, providing both the class prediction and class probabilities. Per sample computation time depends on the number of missing values in a sample, with per sample run time across the validation cohort ranging from 1 to 22 s (approximately 7 h for all 10,376 samples).
