# Supplementary material for: A platform-independent AI tumor lineage and site (ATLAS) classifier
Source: Commun Biol. 2024 Mar 13;7:314. doi: 10.1038/s42003-024-05981-5 (PMC10937974; doi:10.1038/s42003-024-05981-5)
Supplement: Supplementary file 1 — Description of Additional Supplementary Files [file 42003_2024_5981_MOESM1_ESM.docx]

**Description of Additional Supplementary Files**

**File name:** Supplementary Data 1

**Description:** Median Shapley Values per Gene and Sample Class for Cancer Site of Origin and Cancer Lineage. The median Shapley values for each feature per class were obtained from the training data to identify the features with the largest importance in predicting the site of origin and lineage classes. The median value corresponds to the Shapley value for that feature across all samples of that specific class, presenting the top 10 features for each class. To confirm that features were specific to the tumor and not just normal tissue, the top 10 features for each class among correctly predicted metastatic samples is reported as well.
